# Electrocardiographic detection of left atrial enlargement in arterial hypertension: re-calibration against cardiac magnetic resonance

**DOI:** 10.1186/1532-429X-18-S1-Q26

**Published:** 2016-01-27

**Authors:** Jonathan C Rodrigues, Tamas Erdei, Bethannie McIntyre, Amardeep Ghosh Dastidar, Amy E Burchell, Laura E Ratcliffe, Emma C Hart, Julian F Paton, Mark Hamilton, Angus K Nightingale, Nathan E Manghat

**Affiliations:** 1 CMR Unit, NIHR Cardiovascular Biomedical Research Unit, Bristol Heart Institute, Bristol, United Kingdom; 2 Cardionomics Research Group, Bristol Heart Institute, Bristol, United Kingdom

## Background

Both American and European guidelines on the management of arterial hypertension advise that an electrocardiogram (ECG) be routinely performed in all patients with arterial hypertension. The ECG may demonstrate evidence of left atrial enlargement (LAE), which has adverse prognostic implications in hypertension. We sought to determine the accuracy of 5 ECG criteria of LAE in a hypertensive cohort relative to cardiac magnetic resonance (CMR) gold-standard. We also sought to investigate the confounding effect of obesity.

## Methods

Consecutive referrals for CMR (1.5T) from a tertiary hypertension clinic were reviewed. Patients with any concomitant cardiac pathology other than hypertension were excluded. ECGs were assessed, blinded to the CMR data, for:

1) P wave >110 ms

2) P mitrale (notched P wave with inter-peak duration >40 ms)

3) P wave axis <30°

4) Area of negative P terminal force in lead V1 (NPTF-V1) > 40 ms·mm

5) Positive P terminal force in aVL (PPTF-aVL) >0.5 mm

LA volume (LAV), excluding LA appendage and pulmonary vein confluence, was measured at maximum atrial diastole using the biplane area-length method, which has previously been validated against LA short axis cine stack measurements. LAV were indexed to body surface area. LAE was defined as ≥55 ml/m2 (>2 standard deviations above the mean published references value for normal, healthy volunteers). CMR analysis was performed blinded to ECG data. Sensitivity, specificity, positive predictive value, negative predictive value and accuracy were calculated. Area under the receiver operator curve analysis was performed.

## Results

130 patients were included (age: 51.4 ± 15.1 years, 47% male, 51% obese, systolic blood pressure: 171 ± 29 mmHg, diastolic blood pressure: 97 ± 15 mmHg).

**Figure 1:**
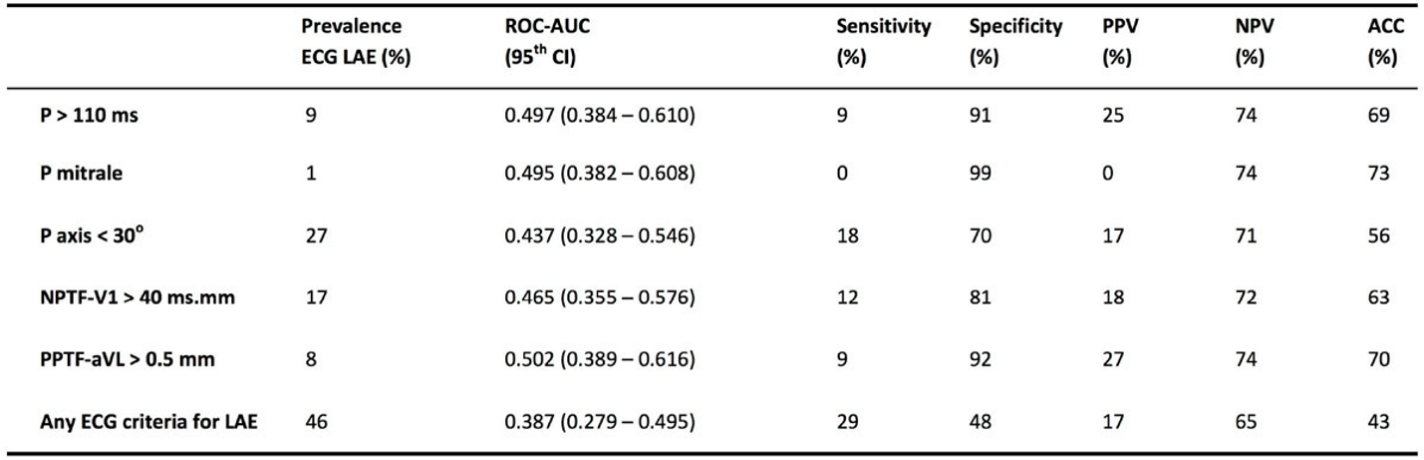
**Diagnostic performance of the various ECG parameters at detecting left atrial enlargement**. (LAE = left atrial enlargement, ROC-AUC = receiver operator curve-area under curve, CI = confidence interval, PPV = positive predictive value, NPV = negative predictive values, ACC = accuracy)

**Figure 2:**
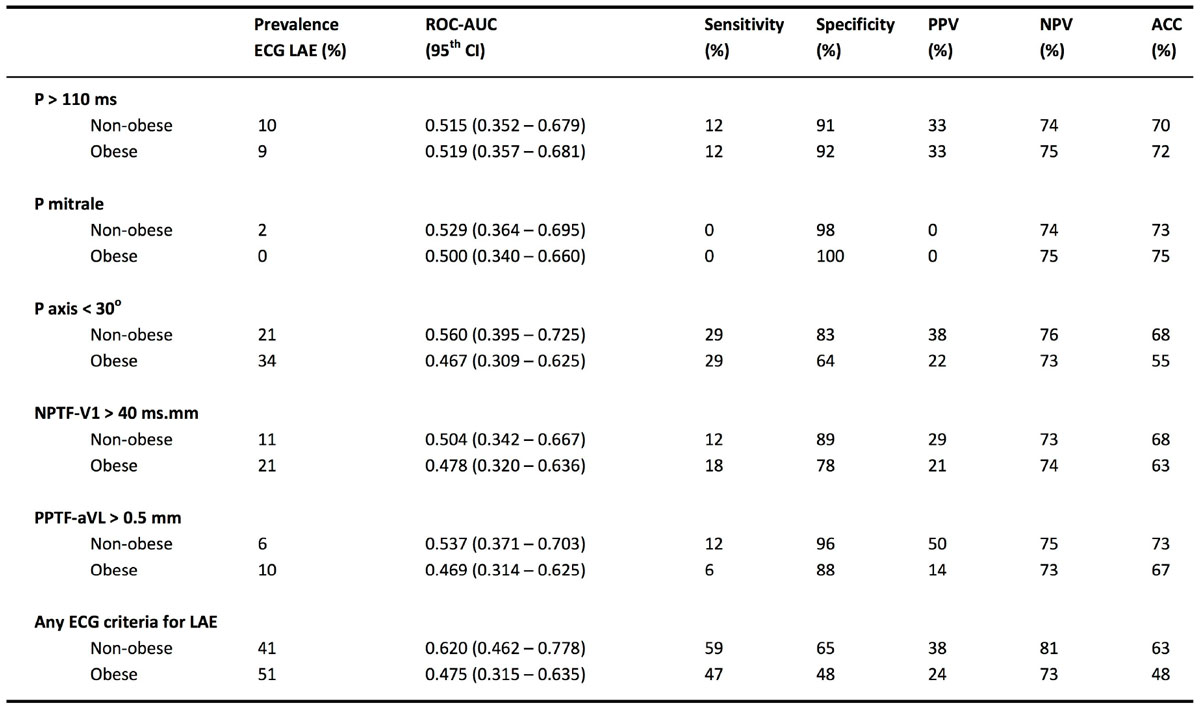
**Gender subgroup analysis of diagnostic performance of the various ECG parameters at detecting left atrial enlargement**. (LAE = left atrial enlargement, ROC-AUC = receiver operator curve-area under curve, CI = confidence interval, PPV = positive predictive value, NPV = negative predictive values, ACC = accuracy)

The prevalence of LAE by CMR was 26% and the prevalence by ECG varied from 1% (P mitrale) to 27% (P axis < 30°), and was 46% when ≥1 ECG LAE criteria were present (Figure 1). There was no significant difference in mean indexed LA volume when ≥1 ECG LAE criterion was present compared to when no ECG LAE criteria were present (47 ± 15 vs 50 ± 15 ml/m^2^, P = 0.235). All the individual ECG LAE criteria were more specific than sensitive (Figure 1), with specificities ranging from 70% (P axis <30°) to 99% (P mitrale). Subgroup analysis by obesity is demonstrated in Figure 2. Obesity attenuated the specificity of most of the individual ECG LAE criteria. Obesity correlated with significant lower specificity (48% vs 65%, P < 0.05) and a trend towards lower sensitivity (59% vs 43%, P = 0.119) when ≥1 ECG criteria of LAE were present

## Conclusions

Individual ECG criteria of LAE in hypertension are specific, but not sensitive, for identifying anatomical LAE, relative to CMR. LAE in hypertension should not be excluded on the basis of the ECG, particularly in obese subjects.

